# Hypothyroidism monitoring and control during the first trimester of pregnancy in Catalonia

**DOI:** 10.3389/fendo.2025.1445977

**Published:** 2025-03-18

**Authors:** Glòria Tena Vivó, Oriol Cunillera Puértolas, Mercè Albareda Riera, Neus Parellada Esquius, Mònica Isidro Albaladejo, Gemma Rodríguez Palomar, Silvia Palmero Aliste, Lluís Vila

**Affiliations:** ^1^ Hospital de Viladecans, Obstetrics and Gynecology Viladecans, Barcelona, Spain; ^2^ Unitat de Suport a la Recerca Metropolitana Sud, Fundació Institut Universitari per a la recerca a l’Atenció Primària de Salut Jordi Gol i Gurina, l’Hospitalet de Llobregat, Catalunya, Spain; ^3^ Endocrinology and Nutrition Division, Hospital de Sant Joan Despí Moisès Broggi, Sant Joan Despí, Spain; ^4^ Institut Català de la Salut Gerència Territorial Metropolitana Sud, Epidemiology and Research, L’Hospitalet de Llobregat, Catalunya, Spain; ^5^ Institut Català de la Salut (ICS), Sexual and Reproductive Primary Health Care, Sant Boi de Llobregat, Spain; ^6^ Servei d’Atenció Primària Delta del Llobregat Gerència Territorial Metropolitana Sud, Pharmacologist, L’Hospitalet de Llobregat, Catalunya, Spain

**Keywords:** hypothyroidism, pregnancy, prevalence, hypothyroidism monitoring, hypothyroidism control

## Abstract

**Objective:**

This study aims to describe hypothyroidism monitoring and control during the first trimester of pregnancy in women with known hypothyroidism in Catalonia.

**Materials and methods:**

Pregnancies registered in primary care in Catalonia between 2014 and 2016 were retrospectively studied. Women with hypothyroidism were selected for the study. Hypothyroidism was defined if, on the date of the last menstrual period (LMP), there was an updated thyroid hormone prescription (code ATC H03AA -levothyroxine) or any of the following active diagnostic codes: ICD-10: E02, E03, E89.0. To evaluate hypothyroidism monitoring and control, thyrotropin (TSH) tests during the first trimester of pregnancy were considered and categorized according to the reference values of each laboratory.

**Results:**

Out of 111,811 pregnancies, 5,574 had known hypothyroidism. TSH was evaluated in 3,158 (56.65%) of them. The TSH values were within the recommended ranges in 1,146 (36.3%) of the cases, being low abnormal in 53 of them (1.7%) and high abnormal in 1,959 (62%).

**Conclusion:**

TSH testing was not evaluated in almost half of the pregnant women with known hypothyroidism in primary care services in Catalonia during the pregnancy first trimester. Among those tested, more than two-thirds had TSH levels outside the target range. This means that it is essential to improve the management of hypothyroidism during the first trimester of pregnancy in Catalonia.

## Introduction

Thyroid normal function is essential during pregnancy to avoid complications and ensure a healthy offspring (1–3). Thyroid dysfunction during gestation is the second most common endocrinopathy after diabetes (4), with more than 3% of pregnancies affected (5).

Pregnancy implies important hormonal changes involving a significant thyroid requirement, which depends on adequate iodine intake and normal thyroid gland function (1, 6). Pregnancy thyroid overloading could lead to new thyroid dysfunctions and worsen the previously known ones (1, 4).

Therefore, to manage thyroid dysfunctions effectively during pregnancy, it is essential to prioritize early detection and implement appropriate treatment for pre-existing conditions (7).

Pregnancy thyroid dysfunction prevalence is estimated for clinical hypothyroidism in 0.3%–1.5% and for subclinical hypothyroidism in 3.5%–18% (1, 7–11). Such variations in findings, especially in the case of subclinical hypothyroidism, are largely influenced by the methodologies applied, TSH evaluation techniques used, and reference values considered (9, 10).

Previous and current clinical guidelines recommend that hypothyroid women who are treated with levothyroxine should have a ≤2.5 mIU/L TSH value during the first trimester of pregnancy (5, 12–14).

There are few clinical practice studies dealing with thyroid dysfunction management during pregnancy (15–18). They report poor control for pregnant people following hypothyroidism treatment, which may affect up to 50% of the cases (15, 16). Studies reporting the control degree of these women in primary healthcare in Catalonia have not been published so far. However, data exist confirming that TSH high levels during the first trimester imply a major risk for adverse obstetrical outcomes in Catalan women (19).

The main objective of the present study is to evaluate hypothyroidism monitoring during the first trimester of gestation in women with previously known hypothyroidism, that is to say, what the adherence to guidelines is by primary healthcare professionals in Catalonia and also to verify if hypothyroidism control reached adequate TSH levels in the tested ones.

## Methodology

A descriptive study of pregnancies in hypothyroid women between 2014 and 2016 was performed. It was based on secondary data from the computerized register of the Primary Health Care Research Information and Development System (SIDIAP, Sistema d’Informació per al Desenvolupament de la Investigació en Atenció Primària) (www.sidiap.org). The pregnancies occurred in Catalonia.

SIDIAP generates a comprehensive amount of medical data providing access to reliable information for primary healthcare research. The data originate mainly from ICS (Institut Català de la Salut) electronic health records and other sources. ICS is the governmental institution that manages the public health system in Catalonia.

The SIDIAP database covers about 80% of the Catalan population with over 7 million inhabitants. SIDIAP includes diagnostic and prescription data from both primary care and hospitals (with the exception of propyl-thyouracil, which is dispensed in hospitals only). The laboratory data are from primary healthcare, not from specialized or private centers.

The analytical results come from 33 laboratories which provide service to primary health centers in Catalonia. A total of 15 different laboratory techniques were used, mainly enzyme-chemiluminescence, electro-chemiluminescence, and immuno-chemiluminescence. The general population reference values (GPRV) for TSH ranged between 0.2–4.2 and 0.34–5.6 mIU/L, and for FT4 they ranged between 0.54–1.24 and 0.78-2.02 ng/dL. During the studied period, three laboratories out of the 33 had reference values adjusted for pregnant people.

The population under study included pregnancies that started between 2014 and 2016 in women with known hypothyroidism and who had at least one follow-up visit to the Sexual and Reproductive Health Programme at the primary healthcare. This program is responsible for antenatal care in Catalonia.

Hypothyroidism was defined if there was an active prescription of thyroid hormones (code ATC H03AA -levothyroxine) or any of the following active diagnoses [ICD-10: E02 subclinical iodine-deficiency hypothyroidism, E03 other hypothyroidism, and E89.0 post-procedural hypothyroidism (total thyroidectomy and post I-131 ablation)] recorded on the date of the last menstrual period (LMP) in the computerized medical records in primary healthcare.

Any hyperthyroidism diagnostic or treatment on the date of the LMP posterior to hypothyroidism evidence was excluded. Multiple pregnancies or pregnancies in women under 16 or above 49 years old were also rejected.

### Sample size and sampling procedures

Pregnant populations with hypothyroidism under levothyroxine treatment have shown TSH testing frequencies between 75.9% and 91.4% (15, 16). No publications studying pregnant women with hypothyroidism disregarding treatment have been found. We could therefore expect lower testing rates in our samples. Conservatively, considering the most demanding 50% percentage, assuming 5% alpha risk and 2% accuracy, 2,396 hypothyroid pregnant people are needed to answer the main goal of the present study.

IDESCAT (Institut d’Estadística de Catalunya) reported 210,943 births between 2014 and 2016 (http://www.idescat.cat/pub/?id=aec,n=259,t=2015). In the SIDIAP database, 134,860 births were registered in the same period. Considering 5% hypothyroidism prevalence (1, 6, 8, 9), around 6,743 cases of hypothyroidism were expected.

### Study variables

According to TSH values during the first trimester of pregnancy, the study main outcomes were defined as follows:

TSH evaluation (yes/no): TSH evaluation undertaken during the first trimester of pregnancy.

Hypothyroidism control (normal: when TSH values are higher or equal to 0.1 mIU/L and lower or equal to 2.5 mIU/L; low abnormal: when TSH values are lower than 0.1 mIU/L; high abnormal according to obstetric guidelines: when TSH values are higher than 2.5 mIU/L; high abnormal according to general population reference values: when TSH values are higher than the GPRV (5, 14).

The following study independent variables were evaluated on the date of the LMP as they are very important health risk factors, especially during pregnancy: age, hypertension (I10, I15), diabetes (E11, E12, E13; E14), obesity (BMI ≥40 kg/m^2^) (E66.8), ischemic heart disease (I20, I21, I22, I23, I24, I25), and hormone therapy (code ATC H03AA -levothyroxine).

### Statistical analysis

All continuous numerical variables were categorized. Study variables were described using absolute and relative frequencies. Chi-square test was used to compare frequencies of TSH evaluation according to independent variables. Distribution homogeneity of TSH levels according to hormonal treatment was tested with chi-square test as well.

A multivariate logistic regression model was fit for TSH evaluation using age, hypertension, diabetes, obesity, morbid obesity, and hormone therapy—interacting with the other factors—as predictors. From this initial model and through a stepwise backward variable selection process based on Akaike Information Criteria, a final reduced model was obtained with those factors which contribute plausibly enough to the model.

For the multivariate logistic regression model, odds ratios are shown. To facilitate interpretation, probabilities of having a TSH evaluation are calculated for all possible profiles according to the regression model.

The R-4.0.3 statistical package for Windows was used.

### Confidentiality and ethical aspects

Confidentiality was ensured since all of the cases in the SIDIAP database are pseudo-anonymous and the established protocol was followed. Therefore, informed consent is not needed.

Study ethical protocol was approved by the Ethical Committee for Clinical Research (Comité Ético de Investigación Clínica CEIC) from IDIAP (Institut Universitari d’Investigació en Atenció Primària) Jordi Gol (P17/113).

## Results

During 2014–2016, 111,811 pregnancies fulfilled the inclusion criteria. From these, 5,574 (4.98%) were pregnant women with known hypothyroidism, with their average age being 32.49 years old (5.52 standard deviation). On the date of the LMP, 31.29% of these women were 35 years old or above, and their prevalence rates for hypertension, diabetes, obesity, morbid obesity, and ischemic heart disease were 1.72%, 1.40%, 14.57%, 0.72%, and 0.02%, respectively. Levothyroxine treatment was followed by 45.96% of them (**Table 1**).

**Table 1:** Characteristics of pregnant women with previously known hypothyroidism and TSH evaluations in the first trimester in accordance with these characteristics.

		Hypothyroid pregnant women (*n* = 5,574)	Hypothyroid pregnant women with TSH evaluation (*n* = 3,158)	*p*-value
Age on the date of the LMP	<35	3,830 (68.71%)	2,189 (57.15%)	0.525
	[35, 40]	1,395 (25.03%)	777 (55.70%)	
	≥40	349 (6.26%)	192 (55.01%)	
Hypertension	No	5,478 (98.28%)	3,112 (56.81%)	0.101
	Yes	96 (1.72%)	46 (47.92%)	
Diabetes	No	5,496 (98.60%)	3,122 (56.80%)	0.077
	Yes	78 (1.40%)	36 (46.15%)	
Obesity	No	4,762 (85.43%)	2,692 (56.53%)	0.676
	Yes	812 (14.57%)	466 (57.39%)	
Morbid obesity	No	5,534 (99.28%)	3,144 (56.81%)	0.009
	Yes	40 (0.72%)	14 (35.00%)	
Ischemic heart disease	No	5,573 (99.98%)	3,157 (56.65%)	1.000
	Yes	1 (0.02%)	1 (100.00%)	
Treatment	Untreated	3,012 (54.04%)	1,421 (47.18%)	<0.001
	Treated with levothyroxine	2,562 (45.96%)	1,737 (67.80%)	

TSH was evaluated in 3,158 (56.65%) of the cases. The median [IQR] TSH values in the subgroup of women who underwent this evaluation was 63 [40, 71], and the mean gestational age at the time of the TSH test was 9 weeks. Bivariate association between age and TSH evaluation was not detected. TSH evaluation was lower among morbid obese women compared to the ones without obesity (35% *versus* 56.81%; *p* = 0.023). TSH evaluation was also lower in diabetic patients (46.15%) compared to the ones without diabetes (56.80%; *p* = 0.08).

TSH was more frequently evaluated in hypothyroid women under treatment (67.80% vs. 47.18%; *p* < 0.001) (**Table 1**).

The TSH figures showed hypothyroidism normal control in 1,146 (36.3%), low abnormal control in 53 (1.7%), and high abnormal control in 1,959 (62%) of the cases. The TSH figures for hypothyroidism control in untreated women were more frequently higher than 2.5 mIU/L (66.3%) compared to the figures obtained in women treated with levothyroxine (58.5%) (**Table 2**).

**Table 2:** TSH control during the first trimester of gestation in hypothyroid pregnant women, globally and according to treatment, on the date of the LMP.

TSH control	Globally (*n* = 3,158)	Untreated (*n* = 1,421)	Treated with levothyroxine (*n* = 1,737)	*p*-value
[<0.1 mIU/L]	53 (1.7%)	8 (0.6%)	45 (2.6%)	<0.001
[0.1-2.5 mIU/L]	1,146 (36.3%)	470 (33.1%)	676 (38.9%)	
[2.5 mIU/L -GPRV]	1,187 (37.6%)	610 (42.9%)	577 (33.2%)	
>GPRV	772 (24.4%)	333 (23.4%)	439 (25.3%)	

GPRV, general population reference values.

No differences were observed between treated and untreated women in terms of frequency of abnormal TSH control according to GPRV.

Obesity and the interaction of diabetes with levothyroxine treatment were excluded during the variable selection process, as they did not provide enough likelihood to the model when other co-variables were present. In the final multivariate model, TSH evaluation in untreated women was inferior compared to women under treatment [OR = 0.37 (0.32, 0.42)]. It was also lower in women over 35 or with any of the covariates included in the model (diabetes, hypertension, and morbid obesity) (**Table 3**). The interaction between these co-variables with levothyroxine treatment showed a dilution of the effect of these co-variables in the probability of recording TSH for untreated patients, except for diabetes. Diabetes kept its OR of 0.55 (0.35, 0.87) both in treated and untreated patients. The estimated probabilities for the different profiles are presented in **Supplementary Material 2**.

**Table 3:** Results from the multivariate logistic regression model explaining TSH evaluations.

	OR (95%CI)	*P*-value		OR interaction with “no treatment”	*P*-value
Intercept	2.47 (2.22, 2.75)	<0.001			
No hormone therapy	0.37 (0.32, 0.42)	<0.001			
Age: [35, 40)	0.75 (0.62, 0.90)	0.002	x	1.31 (1.02, 1.69)	0.038
Age: ≥40	0.67 (0.50, 0.91)	0.010	x	1.45 (0.92, 2.29)	0.111
Hypertension	0.53 (0.31, 0.91)	0.022	x	1.98 (0.86, 4.55)	0.108
Diabetes	0.55 (0.34, 0.87)	0.011			
Morbid obesity	0.28 (0.10, 0.70)	0.008	x	2.11 (0.56, 7.99)	0.266

Final model obtained from Akaike Information Criteria backward stepwise selection of variables. Results are expressed as odds ratios. Intercept stands for the odds of TSH evaluation for the reference individual. The reference individual is a pregnant woman with hypothyroidism, aged under 35, in levothyroxine treatment and without hypertension, diabetes nor morbid obesity. For ease of interpretation, estimated probabilities of having a TSH evaluation for all possible profile combinations are presented in **Supplementary Material**.

## Discussion

This study reveals inadequate control in pregnant women with known hypothyroidism in a high percentage of pregnant women in Catalonia since 43.35% of them did not have a TSH evaluation in primary care. Moreover, among the tested ones, more than 60% were out of the recommended control ranges during gestation.

The prevalence of hypothyroidism in pregnant women observed in this study (4.98%) is lower than that reported in previous papers based on the Spanish general population ((9.1% (20)), but similar to the data published about the European general population by Taylor et al. (21) (0.2%–5.3%). Besides, it is higher than the one mentioned for the USA general population by Chaker et al. with rates between 0.3% and 3.7% (22).

The reported prevalence of hypothyroidism in pregnant women varies across different papers. This prevalence ranges from 0.3% to 1.5% for overt hypothyroidism and from 0.2% to 18% for subclinical hypothyroidism (1, 4, 7). The epidemiology of thyroid disorders is variable because it depends on multiple factors, such as illness definition, different methodology used to measure thyroid hormones, different reference ranges applied, iodine nutritional intake, thyroid autoimmunity, or the target population under study (10).

TSH was evaluated in 56.65% of the 5,574 hypothyroid pregnant women. This means that no TSH test was evaluated for almost half of the hypothyroid pregnant women during their first trimester in primary healthcare. Out of the total, 45.82% were under treatment with levothyroxine. In this group, TSH evaluation was 67.80%. This figure is significantly higher compared to the group without treatment, but it is far below the one recommended in the guidelines. ATA 2011 recommended a TSH test every 4 weeks for all hypothyroid pregnant women under levothyroxine treatment, so this figure is really alarming (5). In other papers, these rates were 57.68% (23), 75.9% (16), and 91.4% (15) for hypothyroid pregnant women under treatment.

It is not possible to compare the current study to that of Taylor et al. with almost 76% TSH evaluation, as the population in Taylor’s study (16) started hypothyroidism treatment just 6 months before pregnancy, nor to that of Granfors et al. (15) where just 163 hypothyroid pregnant women were included in the study. Moreover, in the Swedish (15) study, all TSH tests were available, hospital and primary care ones. In the present study, hospital data are not available, which could explain the difference, since pregnant women with comorbidities are monitored in hospitals in Catalonia. Nonetheless, just 1,030 hypothyroid women out of the total (18.4%) have one comorbidity including obesity, which implies that those pregnancies require hospital control.

Bivariate association between age on the date of the LMP and TSH test evaluation has not been found. Despite that ATA 2011 and 2017 recommend one TSH test in all pregnant women aged over 30 (5, 12), whether or not they have thyroid problems, just 56.66% of the cases with hypothyroidism have a TSH evaluation. If we realized that 66% of the participants were over 30 years old, a TSH test should have been ordered in all of these cases regardless of the diagnosis.

It must be acknowledged that the older the maternal age is, the more risk factors associated with pregnancy could appear, which implies an increasing need for pregnancy hospital controls. This fact could explain why pregnant women with morbid obesity have less TSH evaluations than the ones without morbid obesity (35% vs. 56.81%; *p* = 0.023) and the same occurs for diabetic women, with regard to the non-diabetic ones (46.15% vs. 56.80%; *p* = 0.08). Probably these women have been monitored in hospitals. Data from hospitals are not available in the present study.

TSH evaluation was higher in the treated group compared to the untreated one (55% vs. 44%; *p* < 0.001). It could be speculated that health professionals consider that untreated women have a lower risk, and for this reason, less controls are needed. Nevertheless, untreated women significantly have an out-of-range TSH percentage (*p* < 0.001) (TSH >2.5 mIU/L, 66.3%) with regard to the ones under treatment with levothyroxine (TSH >2.5 mIU/L, 58.5%), which implies an increased risk for these pregnancies (19).

TSH values >2.5 mIU/L were found in 62% in hypothyroid pregnant women. Such a figure is very high but similar to the ones reported in other papers (67%–69.41%) (16, 17). Moreover, in the present study, TSH values >2.5 mIU/L have been found in 58.5% pregnant women under levothyroxine treatment. This figure is slightly higher than the ones reported in other studies [50.9%–55% (15, 17)], although it is worth noting that the above mentioned reports have much lower sample sizes than the one in this study.

All of these data reveal that the recommendations in the guidelines have not been implemented. Clinical guidelines recommend to plan pregnancies and begin or increase levothyroxine treatment depending on previous TSH values when pregnancy starts (5, 12, 14). That is to say, there is a significant discrepancy between the clinical guidelines’ recommendations and daily clinical practice (16).

Even without taking into account the TSH cutoff point of 2.5 mIU/L, as recommended by ATA 2011, a large percentage of pregnant women is above the GPRV. TSH values above the GPRV are very prevalent in pregnant women, regardless of being under treatment or not. Good monitoring must be emphasized since a large number of research has proved a greater risk for pregnancies when the TSH levels are out of the recommended ranges (1–4, 6, 19).

TSH was found to be <0.1 mIU/L in 53 hypothyroid pregnant women during the first trimester (1.7%). Among the ones under levothyroxine treatment, such percentage was 2.6%. All of these women were over-treated, but this figure is lower than the one in the study of Taylor et al. (5.11%) (16). Taylor shows that TSH suppression due to overtreatment for a short period does not lead to worse perinatal results. Despite this, Lemieux et al. (23) report an increased risk of prematurity in over-treated pregnant women.

This study has some limitations. The data used come from registers that are not intended for research. Complete information on thyroid autoimmunity was not available for the whole population studied, consequently limiting the analysis of the results. There is also no direct information about the studied population’s diet, although the last study carried out in Catalonia in the pregnant population showed an adequate iodine intake (24). Some codes might not have been used properly due to human errors or difficulties in selecting the proper ICD-10 code in some cases. In addition, some codes could still be active after the illness’ resolution.

This may explain why 33.05% of women diagnosed with untreated hypothyroidism had a TSH <2.5 mIU/L. Several studies have described spontaneous remissions of subclinical hypothyroidism. The study by Rosario et al. (25), conducted in a group of 142 women between 20 and 70 years of age with subclinical hypothyroidism, shows that after 5 years of follow-up, 36% and 60% had normalized TSH, depending on whether they had positive or negative thyroid autoimmunity. Diez et al. (26) also observed regression of subclinical hypothyroidism in 52% of patients after 32 months of follow-up. Furthermore, the high percentage of women diagnosed with hypothyroidism who have a normal TSH without treatment could also be explained by the criteria used for the diagnosis (9, 10).

ESCA (Enquesta de Salut de Catalunya) (Catalan Health Survey) reveals that 25.4% of Catalan women aged between 15 and 44 had private and public health care coverage in 2014. This figure increased up to 29% during 2015 and 2016. It is not possible to know if some participants had TSH tests in private healthcare.

(https://scientiasalut.gencat.cat/handle/11351/1566/browse?resetOffset=true,type=dateissued,order=DESC,rpp=100).

The relevant strengths in this study are the large sample size and the database used, which covers more than 80% of the Catalan population with more than 7 million inhabitants.

## Conclusion

A TSH test was not evaluated in almost half of pregnant women with previously known hypothyroidism in a primary healthcare in Catalonia during the first trimester of gestation. Besides, among the tested ones, more than 60% had TSH levels out of the recommended ranges.

This study shows that it is essential to improve the management of hypothyroidism during the first trimester of pregnancy in Catalonia. It is crucial that healthcare professionals and pregnant women raise awareness that the controls have to be done earlier and regularly.

It can be concluded that there is an urgent need to improve hypothyroidism monitoring and control during the first trimester in hypothyroid pregnant women in Catalonia.

## Data Availability

Publicly available datasets were analyzed in this study. The data that support the findings of this study are not publicly available on legal or ethical grounds, as they contain information that could compromise the privacy of research participants, but are available from Oriol Cunillera Puértolas upon reasonable request.
