# Supplementary material for: Hypothyroidism monitoring and control during the first trimester of pregnancy in Catalonia
Source: Front Endocrinol (Lausanne). 2025 Mar 18;16:1445977. doi: 10.3389/fendo.2025.1445977 (PMC11958182; doi:10.3389/fendo.2025.1445977)
Supplement: Supplementary file 1 [file Table1.docx]

**Supplementary material 1.** Pregnant women characteristics with previously known hypothyroidism according to treatment, on the date of the LMP.

|  |  | **Untreated (n=3,012)** | **Treated with Levothyroxine (n=2,562)** |  |
| --- | --- | --- | --- | --- |
| Age on the date of the LMP | *<35* | 2,209 (73.34%) | 1,621 (63.27%) | <0.001 |
|  | *[35, 40]* | 663 (22.01%) | 732 (28.57%) |  |
|  | *>=40* | 140 (4.65%) | 209 (8.16%) |  |
| Hypertension |  | 41 (1.36%) | 55 (2.15%) | 0.032 |
| Diabetes |  | 25 (0.83%) | 53 (2.07%) | <0.001 |
| Obesity |  | 447 (14.84%) | 365 (14.25%) | 0.556 |
| Morbid Obesity |  | 20 (0.66%) | 20 (0.78%) | 0.723 |
| Ischemic Heart Disease |  | 1 (0.03%) | 0 (0.00%) | 1.000 |
