# Supplementary material for: Hypothyroidism monitoring and control during the first trimester of pregnancy in Catalonia
Source: Front Endocrinol (Lausanne). 2025 Mar 18;16:1445977. doi: 10.3389/fendo.2025.1445977 (PMC11958182; doi:10.3389/fendo.2025.1445977)
Supplement: Supplementary file 2 [file Table2.docx]

**Supplementary material 2.** Estimated probabilities of having TSH evaluation in primary care according to the multivariate logistic regression model. Multivariate logistic regression model is presented in table 3.

| **Hormone Therapy** | **Age group** | **Hypertension** | **Diabetes** | **Morbid Obesity** | **TSH evaluation probability** |
| --- | --- | --- | --- | --- | --- |
| Under treatment | <35 | No hypertension | No diabetes | No morbid obesity | 0.712 (0.689, 0.733) |
| Under treatment | [35, 40] | No hypertension | No diabetes | No morbid obesity | 0.648 (0.613, 0.682) |
| Under treatment | 40+ | No hypertension | No diabetes | No morbid obesity | 0.624 (0.556, 0.688) |
| Under treatment | <35 | No hypertension | Diabetes | No morbid obesity | 0.576 (0.460, 0.683) |
| Under treatment | <35 | Hypertension | No diabetes | No morbid obesity | 0.566 (0.430, 0.693) |
| Under treatment | [35, 40] | No hypertension | Diabetes | No morbid obesity | 0.503 (0.386, 0.620) |
| Under treatment | [35, 40] | Hypertension | No diabetes | No morbid obesity | 0.493 (0.360, 0.628) |
| No treatment | <35 | Hypertension | No diabetes | No morbid obesity | 0.486 (0.335, 0.640) |
| No treatment | [35, 40] | Hypertension | No diabetes | No morbid obesity | 0.481 (0.330, 0.635) |
| No treatment | 40+ | Hypertension | No diabetes | No morbid obesity | 0.480 (0.316, 0.647) |
| Under treatment | 40+ | No hypertension | Diabetes | No morbid obesity | 0.477 (0.346, 0.610) |
| No treatment | <35 | No hypertension | No diabetes | No morbid obesity | 0.475 (0.454, 0.496) |
| No treatment | [35, 40] | No hypertension | No diabetes | No morbid obesity | 0.470 (0.432, 0.508) |
| No treatment | 40+ | No hypertension | No diabetes | No morbid obesity | 0.469 (0.387, 0.552) |
| Under treatment | 40+ | Hypertension | No diabetes | No morbid obesity | 0.467 (0.329, 0.611) |
| Under treatment | <35 | Hypertension | Diabetes | No morbid obesity | 0.417 (0.264, 0.589) |
| Under treatment | <35 | No hypertension | No diabetes | Morbid obesity | 0.410 (0.214, 0.638) |
| No treatment | <35 | Hypertension | No diabetes | Morbid obesity | 0.360 (0.164, 0.616) |
| No treatment | [35, 40] | Hypertension | No diabetes | Morbid obesity | 0.355 (0.161, 0.611) |
| No treatment | 40+ | Hypertension | No diabetes | Morbid obesity | 0.354 (0.156, 0.619) |
| No treatment | <35 | No hypertension | No diabetes | Morbid obesity | 0.350 (0.175, 0.576) |
| Under treatment | [35, 40] | Hypertension | Diabetes | No morbid obesity | 0.349 (0.211, 0.517) |
| No treatment | [35, 40] | No hypertension | No diabetes | Morbid obesity | 0.345 (0.171, 0.573) |
| No treatment | 40+ | No hypertension | No diabetes | Morbid obesity | 0.344 (0.164, 0.584) |
| No treatment | <35 | Hypertension | Diabetes | No morbid obesity | 0.342 (0.194, 0.529) |
| Under treatment | [35, 40] | No hypertension | No diabetes | Morbid obesity | 0.341 (0.168, 0.571) |
| No treatment | [35, 40] | Hypertension | Diabetes | No morbid obesity | 0.337 (0.190, 0.523) |
| No treatment | 40+ | Hypertension | Diabetes | No morbid obesity | 0.336 (0.183, 0.533) |
| No treatment | <35 | No hypertension | Diabetes | No morbid obesity | 0.332 (0.237, 0.443) |
| No treatment | [35, 40] | No hypertension | Diabetes | No morbid obesity | 0.327 (0.231, 0.441) |
| No treatment | 40+ | No hypertension | Diabetes | No morbid obesity | 0.326 (0.217, 0.459) |
| Under treatment | 40+ | Hypertension | Diabetes | No morbid obesity | 0.325 (0.189, 0.499) |
| Under treatment | 40+ | No hypertension | No diabetes | Morbid obesity | 0.318 (0.151, 0.549) |
| Under treatment | <35 | No hypertension | Diabetes | Morbid obesity | 0.276 (0.120, 0.516) |
| Under treatment | <35 | Hypertension | No diabetes | Morbid obesity | 0.268 (0.112, 0.516) |
| No treatment | <35 | Hypertension | Diabetes | Morbid obesity | 0.236 (0.090, 0.491) |
| No treatment | [35, 40] | Hypertension | Diabetes | Morbid obesity | 0.232 (0.088, 0.486) |
| No treatment | 40+ | Hypertension | Diabetes | Morbid obesity | 0.231 (0.085, 0.493) |
| No treatment | <35 | No hypertension | Diabetes | Morbid obesity | 0.228 (0.095, 0.455) |
| No treatment | [35, 40] | No hypertension | Diabetes | Morbid obesity | 0.224 (0.092, 0.451) |
| No treatment | 40+ | No hypertension | Diabetes | Morbid obesity | 0.223 (0.088, 0.460) |
| Under treatment | [35, 40] | No hypertension | Diabetes | Morbid obesity | 0.222 (0.092, 0.445) |
| Under treatment | [35, 40] | Hypertension | No diabetes | Morbid obesity | 0.215 (0.086, 0.444) |
| Under treatment | 40+ | No hypertension | Diabetes | Morbid obesity | 0.204 (0.082, 0.424) |
| Under treatment | 40+ | Hypertension | No diabetes | Morbid obesity | 0.198 (0.077, 0.419) |
| Under treatment | <35 | Hypertension | Diabetes | Morbid obesity | 0.168 (0.061, 0.386) |
| Under treatment | [35, 40] | Hypertension | Diabetes | Morbid obesity | 0.131 (0.046, 0.320) |
| Under treatment | 40+ | Hypertension | Diabetes | Morbid obesity | 0.119 (0.041, 0.300) |
